# Expression of hormone receptors predicts survival and platinum sensitivity of high-grade serous ovarian cancer

**DOI:** 10.1042/BSR20210478

**Published:** 2021-05-06

**Authors:** Jiahong Tan, Chunyan Song, Daoqi Wang, Yigang Hu, Dan Liu, Ding Ma, Qinglei Gao

**Affiliations:** 1 Department of Obstetrics and Gynecology, Tongji Hospital, Tongji Medical College, Huazhong University of Science and Technology, Wuhan 430030, People’s Republic of China; 2 Cancer Biology Research Center, Tongji Hospital, Tongji Medical College, Huazhong University of Science and Technology, Wuhan 430030, People’s Republic of China; 3 Department of Urology, Tongji Hospital, Tongji Medical College, Huazhong University of Science and Technology, Wuhan 430030, People’s Republic of China

**Keywords:** hormone receptors, ovarian cancer, platinum responsiveness, prediction, survival

## Abstract

High-grade serous ovarian cancer (HGSOC) has abundant expression of hormone receptors, including androgen receptor (AR), estrogen receptor α (ER), and progesterone receptor (PR). The effects of hormone receptors on prognosis of HGSOC were first evaluated in online databases. Their prognostic values were then explored and validated in our inhouse TJ-cohort (92 HGSOC patients) and in a validation cohort (33 HGSOC patients), wherein hormone receptors were detected immunohistochemically. High expression of hormone receptors denoted longer progression-free survival (PFS), overall survival (OS), and platinum-free interval (PFI). Platinum-sensitive patients had higher expression of hormone receptors than their counterparts. Correlation analysis revealed significant positive correlations between hormone receptors expression and survival. AR, ER, and PR had predictive and prognostic values, alone and in combination. By receiver operating characteristic curve (ROC) analysis, co-expression of AR, ER, and PR had an improved predictive performance with an area under the curve (AUC) value of 0.945. Expression of hormone receptors predicts survival and platinum sensitivity of HGSOC. AR, ER, and PR might be feasible prognostic biomarkers for HGSOC by immunohistochemical analysis.

## Introduction

Ovarian cancer is the second leading cause of gynecologic cancer deaths in women around the world, with 152000 deaths annually [1]. For decades, the 5-year survival rate of ovarian cancer remains unchanged at approx. 40% [1]. High-grade serous ovarian cancer (HGSOC) is the most common histological subtype and has aggressive tumor biology [1,2]. Platinum-based chemotherapy has been the standard of care for HGSOC for almost 40 years [3]. Nearly all HGSOC patients will receive platinum-containing regimens as the first-line treatment option [1,4]. However, without pre-determination of platinum responsiveness, 30% of patients have undergone multiple rounds of useless and even toxic treatment [5]. As a highly deadly and heterogeneous disease, subtype-specific biomarkers for the large group of HGSOC patients are urgently needed [6].

Ovarian cancer is partly hormone-dependent [7]. Sex steroids function through their receptors correspondingly [8]. Hormone receptors, including androgen receptor (AR), estrogen receptor α (ER), and progesterone receptor (PR), are considered to be implicated in ovarian carcinogenesis [9–11]. In contrast with breast cancer and prostate cancer, wherein the therapeutic and prognostic roles of AR, ER, and PR are already well established, the prognostic and predictive values of hormone receptors in ovarian cancer are inconsistent and sometimes contradictory [6,12]. Although AR, ER, and PR are widely expressed in every histologic subtype of ovarian cancer, their distribution varies significantly by histology [8]. AR positivity is found to be higher in the serous subtype [13]. The reported frequency of AR expression in HGSOC ranges from 20 to 50% [8,11]. Studies examining the role of AR in HGSOC have been relatively few [8]. ER is expressed in more than half of ovarian cancer, approximately 80–95% HGSOC express ER [14]. Results concerning ER expression and prognosis of HGSOC are controversial [15–18]. The positive rate of PR in HGSOC varies from 20 to 60% [8,19]. PR has been recognized as a good prognostic biomarker for HGSOC [20], whereas conflicting results have been reported regarding its effect on treatment behaviors [21–24]. Concerning prognosis, AR was reported to interact with ER [25]. There was a positive correlation between ER and PR as well [2].

In the present study, we explored the relationship among these three hormone receptors and prognosis of HGSOC. The effects of AR, ER, and PR on survival and platinum sensitivity of ovarian cancer were evaluated using online databases. To further confirm the results, the prognostic values of hormone receptors were explored in our in-house cohorts. AR, ER, and PR might be feasible biomarkers to predict prognosis of HGSOC.

## Methods

### Patients and clinical samples

In the discovery stage, a retrospective analysis including 92 HGSOC patients (TJ-cohort) was performed to examine the relationship between ER/PR expression and survival and platinum sensitivity. ER/PR expression of these patients was retrieved from the medical records. Patient characteristics were summarized in Table 1. Among these 92 patients, 85 of them have received platinum-based chemotherapy after initial debulking surgery. Patient characteristics were shown in Table 2. In the validation phase, the relationship between AR/ER/PR expression and prognosis was analyzed in a cohort containing 33 HGSOC patients. For the validation cohort, formalin-fixed paraffin-embedded sections were used to determine the expression of hormone receptors. Patient characteristics were listed in Table 3. Platinum resistance and sensitivity were defined as recurrence within 6 months or after more than 6 months [4]. Written informed consent of patients is routinely requested in our institution for data and sample collection for research purpose. The study was performed in accordance with the World Medical Association Declaration of Helsinki and supervised by the Ethics Committee of Tongji Medical College (Reference Number: S267). All patients were hospitalized at the Gynecology Department of Tongji Medical College affiliated Tongji Hospital, Huazhong University of Science and Technology.

**Table 1:** Clinicopathological characteristics of patients in TJ-cohort

Characteristics	Total patients	ER positive		ER negative		*P*-value	PR positive		PR negative		*P*-value
	(*n*=92)	(*n*=66)		(*n*=26)			(*n*=46)		(*n*=46)		
	Number	Number	%	Number	%		Number	%	Number	%	
Age at diagnosis						0.2591					0.2105
≤50 years	44	34	51.52%	10	38.46%		19	41.30%	25	54.35%	
>50 years	48	32	48.48%	16	61.54%		27	58.70%	21	45.65%	
FIGO stage						0.1210					0.3647
I	9	9	13.64%	0	0.00%		7	15.22%	2	4.35%	
II	16	9	13.64%	7	26.92%		7	15.22%	9	19.57%	
III	59	43	65.15%	16	61.54%		28	60.87%	31	67.39%	
IV	8	5	7.57%	3	11.54%		4	8.69%	4	8.69%	
Histologic type	HGSOC	HGSOC		HGSOC			HGSOC		HGSOC		
Ascites						0.5421					0.3435
Yes	60	42	63.64%	18	69.23%		27	58.70%	33	71.74%	
No	17	14	21.21%	3	11.54%		11	23.91%	6	13.04%	
Unknown	15	10	15.15%	5	19.23%		8	17.39%	7	15.22%	
Chemotherapy						0.0841					0.2381
Platinum-based	85	59	89.39%	26	100.00%		41	89.13%	44	95.65%	
other	7	7	10.61%	0	0.00%		5	10.87%	2	4.35%	

Abbreviation: FIGO, International Federation of Gynecology and Obstetrics.

**Table 2:** Clinicopathological characteristics of patients receiving platinum-containing chemotherapy in TJ-cohort

Characteristics	Total patients	ER positive		ER negative		*P*-value	PR positive		PR negative		*P*-value
	(*n*=85)	(*n*=59)		(*n*=26)			(*n*=41)		(*n*=44)		
	Number	Number	%	Number	%		Number	%	Number	%	
Age at diagnosis						0.2313					0.2278
≤50 years	41	31	52.54%	10	38.46%		17	41.46%	24	54.55%	
>50 years	44	28	47.46%	16	61.54%		24	58.54%	20	45.45%	
FIGO stage											
I	8	8	13.56%	0	0.00%	0.1707	6	14.63%	2	4.55%	0.4542
II	16	9	15.25%	7	26.92%		7	17.07%	9	20.45%	
III	53	37	62.71%	16	61.54%		24	58.54%	29	65.90%	
IV	8	5	8.48%	3	11.54%		4	9.76%	4	9.10%	
Histologic type	HG-SOC	HGSOC		HGSOC			HGSOC		HGSOC		
Ascites						0.6176					0.4835
Yes	55	37	62.71%	18	69.23%		24	58.54%	31	70.45%	
No	15	12	20.34%	3	11.54%		9	21.95%	6	13.64%	
Unknown	15	10	16.95%	5	19.23%		8	19.51%	7	15.91%	

Abbreviation: FIGO, International Federation of Gynecology and Obstetrics.

**Table 3:** Clinicopathological characteristics of patients in the validation cohort

Characteristics	AR positive		AR negative		*P*-value	ER positive		ER negative		*P*-value	PR positive		PR negative		*P*-value
	(*n*=23)		(*n*=10)			(*n*=26)		(*n*=7)			(*n*=21)		(*n*=12)		
	Number	%	Number	%		Number	%	Number	%		Number	%	Number	%	
Age at diagnosis					0.5615					0.9792					0.3921
≤50 years	9	39.13%	5	50.00%		11	42.31%	3	42.86%		9	42.86%	7	58.33%	
>50 years	14	60.87%	5	50.00%		15	57.69%	4	57.14%		12	57.14%	5	41.67%	
FIGO stage					0.5822					0.7912					0.3698
II	6	26.09%	1	10.00%		6	23.08%	1	14.29%		6	28.57%	1	8.33%	
III	15	65.22%	8	80.00%		18	69.23%	5	71.42%		13	61.90%	10	83.34%	
IV	2	8.69%	1	10.00%		2	7.69%	1	14.29%		2	9.53%	1	8.33%	
Histologic type	HGSOC		HGSOC			HGSOC		HGSOC			HGSOC		HGSOC		
Ascites					0.9801					0.4155					0.7746
Yes	16	69.57%	7	70.00%		19	73.08%	4	57.14%		15	71.43%	8	66.67%	
No	7	30.43%	3	30.00%		7	26.92%	3	42.86%		6	28.57%	4	33.33%	

Abbreviation: FIGO, International Federation of Gynecology and Obstetrics.

### Immunohistochemistry

Immunohistochemical analysis was performed as reported previously [26]. Briefly, formalin-fixed paraffin-embedded tissue sections were deparaffinized and rehydrated. After heat-induced antigen retrieval (TE buffer pH 9.0, G1203, ServiceBio, China), the slides were analyzed using an Avidin–Biotin Complex Vectastain Kit (SP9001, ZSGB-Bio, China) as per the manufacturer’s instructions. Primary antibodies, including AR (1:100, 22089-1-AP, Proteintech, China), ER (1:250, 21244-1-AP, Proteintech), and PR (1:100, 25871-1-AP, Proteintech) were used according to the manufacturer’s guidelines. After incubation with a peroxidase-conjugated secondary antibody (Rabbit, 1:100, ServiceBio), the slides were detected using diaminobenzidine (G1212-200T, ServiceBio). Finally, the samples were counterstained with Hematoxylin. The percentage of positive tumor cells was determined in accordance with the pathologic report of Tongji Hospital. All slides were examined by two investigators, who were blinded to all clinicopathologic variables.

### Online database analysis

The Human Protein Atlas (https://www.proteinatlas.org) was used to analyze the expression of hormone receptors in various cancers. Survival analysis was performed using KM-plotter (https://kmplot.com/analysis/). An online cancer microarray database, Oncomine (https://www.oncomine.org/resource/main.html), was used to detect hormone receptors expression under different platinum responsiveness. Protein–protein interaction was analyzed using STRING (https://string-db.org).

### Statistical analysis

Data were analyzed and plotted using GraphPad Prism 7 (GraphPad Software, San Diego, CA) and presented as the mean ± SD. Differences between groups were compared using two-sided Student’s *t* test unless otherwise indicated. Chi-squared (or Fisher’s exact) test was used to examine the relationship between hormone receptors’ expression and clinicopathologic variables of HGSOC. The value of area under the curve (AUC) was calculated from the receiver operating characteristic curve (ROC) using SPSS (version 25.0). For correlation analysis, Pearson’s correlation test was used to assess the statistical significance. By Kaplan–Meier survival plot, Log-rank test was used to evaluate the differences. Significance was assessed at the level of *P*<0.05.

## Results

### Expression of hormone receptors denotes survival of ovarian cancer

**Figure 1:**
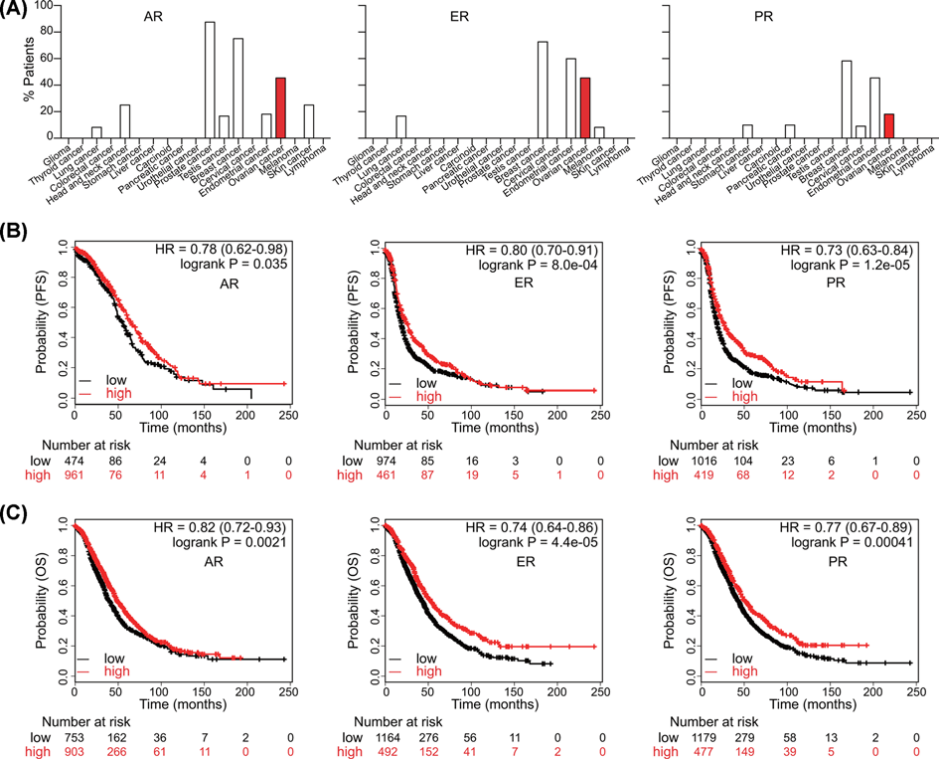
The prognostic role of hormone receptors in ovarian cancer (**A**) Expression of hormone receptors in various cancers. Survival curves for (**B**) PFS and (**C**) OS of AR, ER, and PR (Log-rank test) in ovarian cancer.

To determine the role of hormone receptors in ovarian cancer, we analyzed The Human Protein Atlas to profile their expression (Figure 1A). A total of 45.5% of ovarian cancer cases archived in the database expressed AR, ranking third among all the tumors surveyed. Approximately half of ovarian cancers expressed ER, taking the third place. One-fifth of ovarian cancer patients expressed PR, having the third highest expression. By survival analysis, we analyzed the effects of hormone receptors on survival. High expression of either hormone receptor predicted better progression-free survival (PFS: AR, hazard ration (HR) = 0.78, *P*=0.035; ER, HR = 0.80, *P*=8.0e-04; PR, HR = 0.73, *P*=1.2e-05) (Figure 1B) and overall survival (OS: AR, HR = 0.82, *P*=0.0021; ER, HR = 0.74, *P*=4.4e-05; PR, HR = 0.77, *P*=0.00041) (Figure 1C).

### Expression of hormone receptors suggests platinum sensitivity of ovarian cancer

**Figure 2:**
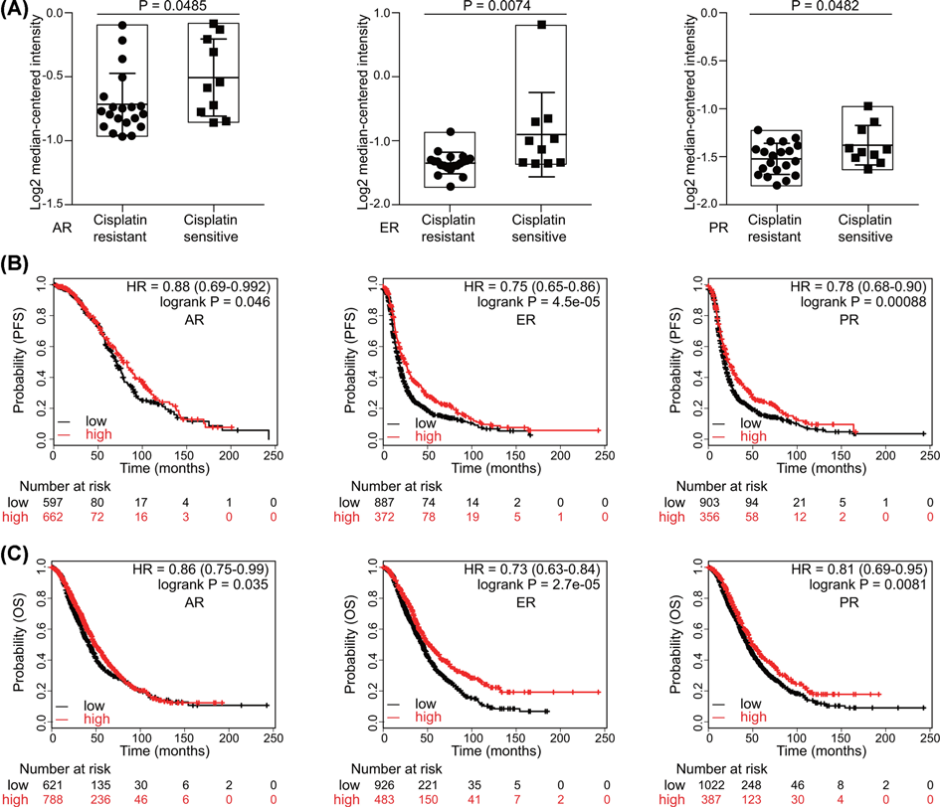
The predictive value of hormone receptors for platinum sensitivity in ovarian cancer (**A**) Expression of hormone receptors in cisplatin-sensitive and cisplatin-resistant ovarian cancer cell lines in Oncomine (Student’s *t* test). Effects of AR, ER, and PR on (**B**) PFS and (**C**) OS in ovarian cancer patients receiving platinum-containing chemotherapy (Log-rank test).

The relationship between hormone receptors and platinum sensitivity was explored in Oncomine. Thirty ovarian cancer cell lines were categorized into two groups according to their response to cisplatin, and then the expression of hormone receptors was compared between the cisplatin-sensitive group and the cisplatin-resistant group. Remarkably, cisplatin-sensitive group expressed higher levels of hormone receptors (AR, *P*=0.0485; ER, *P*=0.0074; PR, *P*=0.0482) (Figure 2A). To further confirm the role of hormone receptors, we then extracted the patients, who had received platinum-containing chemotherapy, for survival analysis. High expression of hormone receptors denoted longer PFS (AR, HR = 0.88, *P*=0.046; ER, HR = 0.75, *P*=4.5e-05; PR, HR = 0.78, *P*=0.00088) (Figure 2B) and OS (AR, HR = 0.86, *P*=0.035; ER, HR = 0.73, *P*=2.7e-05; PR, HR = 0.81, *P*=0.0081) (Figure 2C).

### Expression of ER and PR affects survival in TJ-cohort

**Figure 3:**
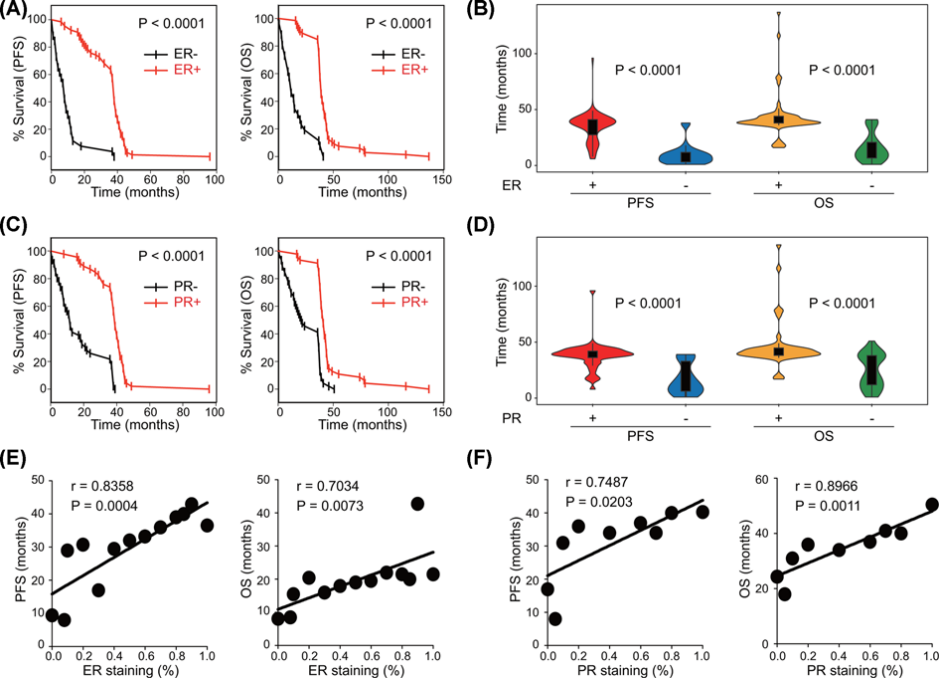
Expression of ER and PR correlates with survival in TJ-cohort (**A**) Survival curves and (**B**) violin plots of PFS and OS in TJ-cohort according to ER expression (Log-rank test and Student’s *t* test). (**C**) Survival curves and (**D**) violin plots of PFS and OS in TJ-cohort according to PR expression (Log-rank test and Student’s *t* test). Correlation analyses between hormone receptors expression and the mean survival time in patients with the same level of expression were performed. (**E**) Correlation analysis of ER expression with the mean PFS and OS of the same ER expression in TJ-cohort (Pearson’s correlation test). (**F**) Correlation analysis of PR expression with the mean PFS and OS of the same PR expression in TJ-cohort (Pearson’s correlation test).

The effects of hormone receptors on survival were further analyzed in our in-house TJ-cohort (92 HGSOC cases) retrospectively. Since AR is not routinely included in medical records, we focused on ER and PR at this discovery stage. In TJ-cohort, there was no significant difference of ER and PR expression among different tumor stages (Supplementary Figure S1A,B). Patients with positive ER expression had significantly longer PFS and OS (Figure 3A,B). Similarly, patients with positive PR expression also had better survival (Figure 3C,D). To further clarify the association between hormone receptors’ expression and patients’ survival, we performed correlation analysis between expression levels and the mean survival time in patients with the same level of expression. Consistently, there was a positive correlation between ER expression levels and mean survival times (PFS, r = 0.8358, *P*=0.0004; OS, r = 0.7034, *P*=0.0073) (Figure 3E). PR expression was also positively related to mean patients’ survival times (PFS, r = 0.7487, *P*=0.0203; OS, r = 0.8966, *P*=0.0011) (Figure 3F).

### Expression of ER and PR indicates platinum sensitivity in TJ-cohort

**Figure 4:**
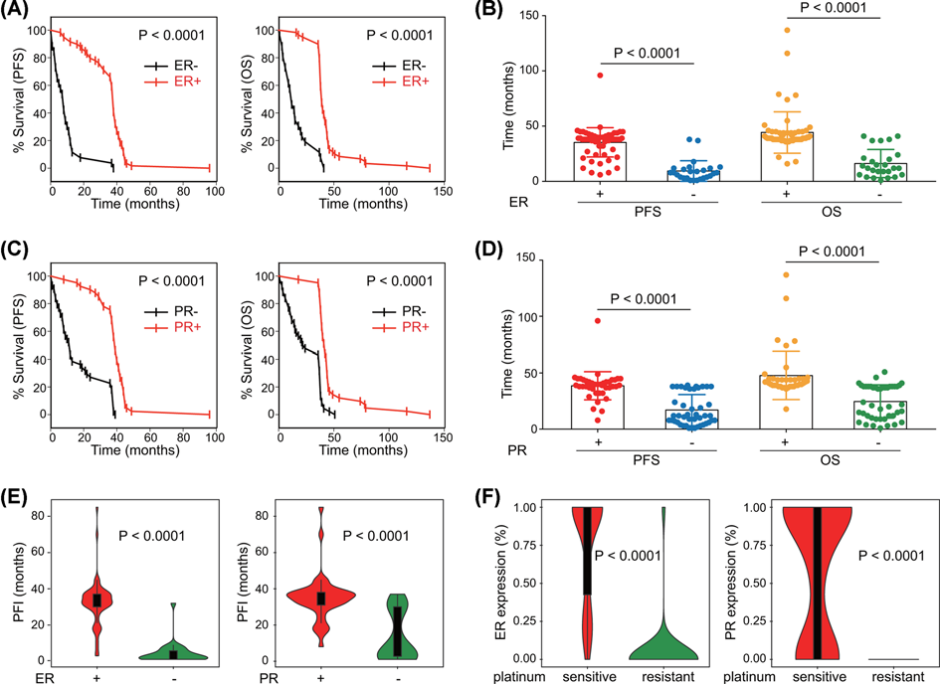
Expression of ER and PR suggests platinum sensitivity in TJ-cohort Patients receiving first-line platinum-based chemotherapy in TJ-cohort were included. (**A**) Survival analysis and (**B**) comparison of PFS and OS according to ER expression (Log-rank test and Student’s *t* test). (**C**) Survival analysis and (**D**) comparison of PFS and OS according to PR expression (Log-rank test and Student’s *t* test). (**E**) Comparison of PFI based on ER or PR expression (Student’s *t* test). (**F**) Comparison of ER and PR expression between platinum-sensitive and platinum-resistant patients (Student’s *t* test).

The relationship between hormone receptors expression and platinum sensitivity was also explored in TJ-cohort. Patients receiving first-line platinum-containing chemotherapy were included for further analysis. Similar results were obtained. A survival benefit was observed in ER-positive patients (Figure 4A,B). The lack of PR expression suggested poorer survival (Figure 4C,D). Platinum-free interval (PFI), an indicator of platinum sensitivity, was calculated from the date of the last platinum-based regimen to the date of recurrence, or defined as 0 month for patients with primary resistance [27]. ER-positive patients and PR-positive patients had significantly longer PFI, implying higher platinum sensitivity (Figure 4E). We then divided the patients into two groups according to their platinum responsiveness. The sensitive group had higher expression of ER and PR than the resistant group (Figure 4F).

### Hormone receptors represent prognostic biomarkers in validation

**Figure 5:**
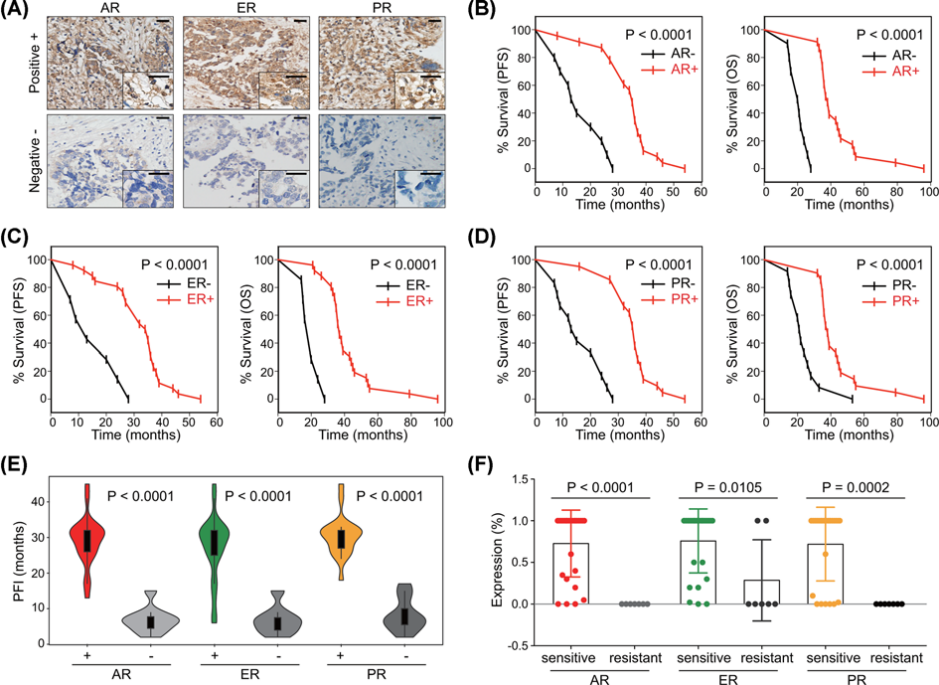
Validation of prognostic values of hormone receptors Expression of AR, ER, and PR was detected using immunohistochemical analysis. (**A**) Representative images of positive and negative staining were shown (bar, 25 µm). Survival curves of PFS and OS in the validation cohort based on the expression of (**B**) AR, (**C**) ER, and (**D**) PR (Log-rank test). (**E**) Violin plot of PFI in the validation cohort (Student’s *t* test). (**F**) Comparison of hormone receptors expression between platinum-sensitive and platinum-resistant groups in the validation cohort (Student’s *t* test).

To further confirm the effects of hormone receptors on the prognosis of ovarian cancer, we employed a validation cohort containing 33 HGSOC patients. Immunohistochemical staining was performed to assess the expression of these three hormone receptors (Figure 5A). Positive expression of hormone receptors denoted more favorable survival (Figure 5B–D). Patients with negative expression of hormone receptors had shorter PFI (Figure 5E). When patients were subdivided into the platinum-sensitive and platinum-resistant groups, the sensitive subgroup had significantly higher expression of hormone receptors (AR, *P*<0.0001; ER *P*=0.0105; PR, *P*=0.0002) (Figure 5F).

### Expression of hormone receptors predicts survival and platinum sensitivity

**Figure 6:**
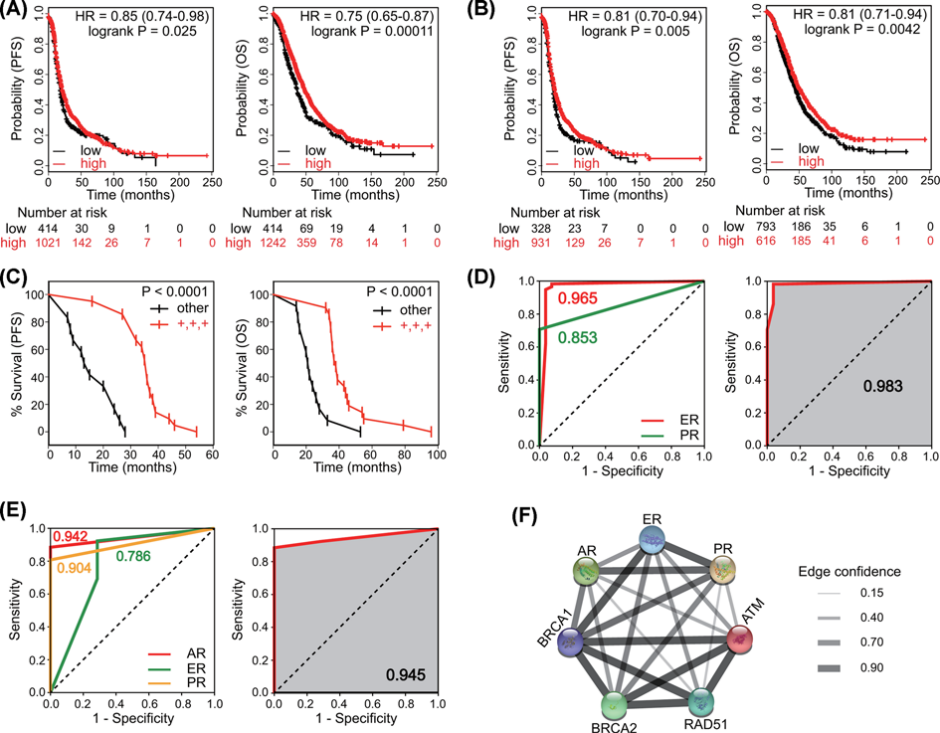
Expression of hormone receptors predicts survival and platinum sensitivity in ovarian cancer In KM-plotter, survival analysis of AR, ER, and PR in combination in (**A**) all patients and (**B**) platinum-treated patients was performed (Log-rank test). Patients in the validation cohort were subdivided into two groups. Patients with triple-positive expression of AR, ER, and PR were grouped together and the remaining patients constituted the other group. (**C**) Survival analysis was performed in these two groups (Log-rank test). (**D**) ROC curves for ER and PR alone and in combination in TJ-cohort. (**E**) ROC curves for AR, ER, and PR alone and in combination in the validation cohort. (**F**) Protein–protein interaction network of hormone receptors and some important DNA damage repair proteins.

Joint analysis of these three receptors was also performed. The combined prognostic value of AR, ER, and PR was significant (PFS, HR = 0.85, *P*=0.025; OS, HR = 0.75, *P*=0.00011) (Figure 6A). For patients receiving platinum-containing chemotherapy, the combination of AR, ER, and PR suggested better PFS (HR = 0.81, *P*=0.005) and OS (HR = 0.81, *P*=0.0042) (Figure 6B). We then subdivided the patients of the validation cohort. Patients with triple-positive expression of AR, ER, and PR were grouped together and the remaining patients constituted the other group. Patients with co-expression of AR, ER, and PR had better PFS (*P*<0.0001) and OS (*P*<0.0001) than those with either negative receptor expression (Figure 6C).

To evaluate the predictive values of hormone receptors expression on platinum sensitivity, ROC curve analysis was performed. In TJ-cohort, the expression of ER or PR alone already had a high predictive value (ER, AUC = 0.965; PR, AUC = 0.853), the combination of both had an even higher predictive value (AUC = 0.983) (Figure 6D). In the validation cohort, AR expression was also analyzed. Co-expression of AR, ER, and PR had an improved predictive performance (AUC = 0.945) (Figure 6E). In STRING, we found a protein–protein interaction between hormone receptors and some important DNA damage repair proteins, including BRCA1, BRCA2, RAD51, and ATM (Figure 6F). Since platinum functions through exacerbating DNA damage [1], it might present the potential mechanism underlying the effects of hormone receptors on platinum sensitivity.

## Discussion

There have been significant research interests in the clinical impacts of hormone receptors on ovarian cancer, concerning both patients’ survival and drug responsiveness [28]. Here, we found that the expression of AR, ER, and PR predicted survival and platinum sensitivity of ovarian cancer. The prognostic values of hormone receptors were confirmed in our in-house HGSOC cohorts, wherein their expression was detected immunohistochemically. Expression of hormone receptors could be exploited to predict survival and platinum sensitivity of HGSOC.

Few biomarkers for prognosis of ovarian cancer have been established owing to the inherent heterogeneity [20]. When all histologic types are combined in a study, the subtype-specific associations will become obscured [20]. Moreover, the expression of AR, ER, and PR among different subtypes of ovarian cancer varies significantly [8]. In this study, we focused on HGSOC and demonstrated that hormone receptors predicted survival and platinum sensitivity of HGSOC. Limited by the data accessibility, we were unable to ascertain the clinicopathological diagnosis of all patients in online survival analysis and had to take all ovarian cancers as a whole. However, the effects of hormone receptors on survival and platinum sensitivity were confirmed in our inhouse cohorts, which contained only HGSOC patients. AR can act as either an oncogene or a tumor suppressor in ovarian cancer [29]. AR was reported to mediate taxol resistance and affect survival of ovarian cancer [30]. Some research revealed that the prognostic value of AR was associated with the length of its CAG repeats and the ethnic origins [31,32]. In this study, we found that AR positivity predicted a survival benefit and platinum sensitivity. Platinum agents are the most widely used therapeutic option in the clinic, so we proposed that AR expression could be a favorable prognostic factor. ER is well-established as cancer associated and expresses in a large fraction of HGSOC [9]. ER expression was reported to be a potential efficacy indicator of endocrine therapy [33]. ER has a role in governing genome stability and affecting homologous recombination repair of ovarian cancer cells [34]. Taken our findings into account, we have reasons to believe that ER expression is a positive predictor of HGSOC prognosis. BRCA1 directly interacts with PR [35], and ATM mutates frequently in PR-positive cancers [36]. These findings suggested the prognostic value of PR. In the present study, PR expression predicted a favorable survival of HGSOC and indicated platinum sensitivity as well. Co-expression of hormone receptors improved the predictive value. Furthermore, these three hormone receptors formed a regulatory network with important DNA damage repair proteins, underlying their effect on platinum responsiveness. Therefore, the expression of hormone receptors could be exploited as prognostic predictors of HGSOC.

Prediction of treatment responses makes sense for HGSOC [10]. For HGSOC patients, treatment regimens are scheduled based on platinum sensitivity [37,38]. However, nearly 20% of patients are inherently resistant to platinum agents [1]. Moreover, improper platinum dosage is toxic and may import resistance to other drugs [3,4]. Some gene profile-based techniques combined with sequencing, microarrays, and PCR were developed to predict prognosis and drug responsiveness of ovarian cancer, but their application had limitations [38,39]. Moreover, detection of protein expression can be more effective than gene screening. As a simple and cost-effective method, immunohistochemical staining has been widely used to evaluate the expression of proteins [40]. When measured immunohistochemically, ER and PR were reported to be more powerful in prediction [20,40,41]. Detection of hormone receptors could help clinicians to identify patients who will actually benefit from platinum agents and refine therapeutic regimens for patients. By immunohistochemical analysis, we evaluated the proportion of positive tumor cells in alignment with clinical practice, without taking the staining intensity of positive nuclei into consideration, which may confound the results. We will integrate both staining proportion and intensity in our future studies. Together, immunohistochemical analysis of hormone receptors provides a feasible approach to predict prognosis of HGSOC.

ER and PR are generally included in the pathologic examination of ovarian cancer, but not AR. Owing to the retrospective nature of the analysis, we were unable to assess the effects of AR on survival and platinum sensitivity in TJ-cohort. Nevertheless, the results from online databases and the validation cohort helped illustrate the prognostic value of AR. Another limitation of the present study is the small sample size. The prognostic values of hormone receptors for HGSOC and other ovarian cancer subtypes warrant further studies in large-scale, multicenter prospective cohorts.

In summary, expression of hormone receptors predicts survival and platinum sensitivity of HGSOC patients. AR, ER, and PR are feasible prognostic biomarkers for HGSOC by immunohistochemical analysis. Hormone receptor-based classification could help stratify patients and guide precision medicine.

## Supplementary Material

Supplementary Figure S1

## Data Availability

All data generated or analyzed during the present study are included in this published article.

## Abbreviations

AR: androgen receptor
AUC: area under the curve
ER: estrogen receptor α
HGSOC: high-grade serous ovarian cancer
HR: hazard ratio
OS: overall survival
PFI: platinum-free interval
PFS: progression-free survival
PR: progesterone receptor
ROC: receiver operating characteristic
