# Supplementary material for: Expression of hormone receptors predicts survival and platinum sensitivity of high-grade serous ovarian cancer
Source: Biosci Rep. 2021 May 6;41(5):BSR20210478. doi: 10.1042/BSR20210478 (PMC8112847; doi:10.1042/BSR20210478)
Supplement: Supplementary Figure S1 [file BSR-2021-0478_supp.pdf]

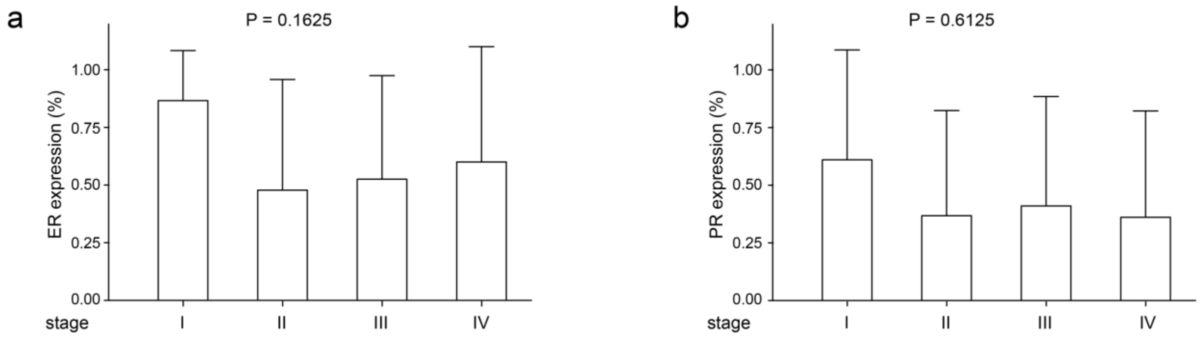

Supplementary Figure s1. Comparison of ER and PR expression among different tumor stages in TJ-cohort. (a) AR expression was compared among different tumor stages in TJ-cohort (one-way ANOVA). (b) PR expression was compared among different tumor stages in TJ-cohort (one-way ANOVA).
